# Wholegrain Food Acceptance in Young Singaporean Adults

**DOI:** 10.3390/nu9040371

**Published:** 2017-04-08

**Authors:** Jia En Neo, Iain A. Brownlee

**Affiliations:** Human Nutrition Research Centre, School of Agriculture, Food & Rural Development, Newcastle University, 172A Ang Mo Kio Ave 8, Singapore 567739, Singapore; jiaennje@hotmail.com

**Keywords:** whole grains, wholegrain foods, healthy eating, young adults

## Abstract

Previous epidemiological evidence suggests that habitual consumption of whole grains is associated with reduction of disease risk. While wholegrain food consumption appears to be increasing in Singapore, it is still low, with more infrequent consumption noted in younger Singaporeans. Therefore, the primary objective of this study is to determine the knowledge of whole grains and barriers to consumption of wholegrain foods. Thirty participants (age range 21–26 year, 19 females) took part in two focus groups separated by a 2-week period in which participants trialled a range of wholegrain foods. Barriers towards whole grain consumption and experiences of products during this familiarization period were discussed during the focus groups and knowledge of whole grains was assessed by questionnaire. Potential barriers such as personal factors, product-specific factors and external factors were identified with sensory and habitual being stronger barriers. The whole grain familiarization period did not alter the taste expectations of the consumers but it did manage to increase acceptance for four of the wholegrain products tested (muesli, cookies, granola bars and wholewheat pasta). These findings suggest existing barriers to wholegrain food consumption should be considered by public health agencies and manufacturing companies.

## 1. Introduction

Whole grains consist of the intact, ground, cracked or flaked fruit of the grains where the primary components (bran, germ, endosperm) are retained within their natural ratio [1]. The putative health enhancing properties of whole grains are attributed to the presence of nutrients and bioactive compounds which are mainly found in the bran and germ [2]. Consistent observational evidence associates habitual consumption of wholegrain foods with a wide range of positive impacts on health such as a reduced risk of diabetes, obesity, cardiovascular disease (CVD) and cancer [3,4].

Singapore is a highly developed island state where food is an important cultural element [5]. The prevalence of chronic diseases associated with low habitual intake of wholegrain foods in Singaporean adults’ aged 18 to 64 years has risen rapidly from 9 to 11.3% (for diabetes) and 6 to 10.8% (for obesity) between 1998 and 2010 respectively [6]. In Singapore, habitual intake of whole grains has increased but still falls well short of recommended intake. While the percentage of adults consuming more than one serving of whole grains a day appears to have risen from 8.4% in 2004 to 27.0% in 2010, younger adults aged 18 to 29 an average consumed over 50% less whole grains than those aged 50 and above [7]. Current public health strategies are targeted at increasing habitual intake of wholegrain foods, particularly through the provision of out-of-home wholegrain alternatives [8] and through use of a Healthier choice symbol for food products [9].

Individuals often face barriers when they attempt to modify their food choice. However it has been suggested that health-related behaviours like increased whole grain consumption would be better encouraged by minimizing perceived barriers and increasing perceived susceptibility, severity and benefits to the new eating behaviour [10]. Therefore, barriers to whole grains must be identified and minimized to encourage consumption of whole grains. Previous research had reported taste, texture, high cost, convenience and limited availability to be the major barriers towards sustainable whole grain intake [11] but that inclusion of wholegrain foods within the diet resulted in higher elective intake following a dietary intervention study [12]. Other factors such as longer preparation time, difficulty in dietary incorporation of whole grains when eating out, personal and other household preferences, lack of knowledge of wholegrain foods, and limited knowledge of health benefits have also been noted [13].

Worldwide, there still appears to be a limited intake of whole grain despite evidence of associations with improved health outcomes. Key to increasing whole grain intake is consumers’ attitudes and perception in relation to these products [11].While there is consistent evidence to support an existence of barriers to whole grain consumption, it is possible that these barriers could be overcome by dietary exposure to, or familiarization with, wholegrain foods. Hence, the aims of this study are to explore consumers’ perception of and potential barriers towards wholegrain foods in a population group with low habitual whole grain intake (i.e., young Singaporean adults) through the use of focus groups either side of a familiarization period.

## 2. Materials and Methods

### 2.1. Participant Recruitment

Ethical approval for this study was obtained from Newcastle University Faculty of Science, Agriculture and Engineering Ethics Committee (project identification code 12-BRO-020, approved on the on the 9 October 2012) in accordance to the Declaration of Helsinki. A convenience sample of 30 young adults (*n* = 19 females) was recruited from the Newcastle University campus in Singapore through local advertisement and word of mouth. The ideal number of participants involved within a study group has previously been suggested to be 10–12 to help facilitate interaction between participants [14] and similar size focus groups have been previously used in studies evaluating consumer perceptions towards foods [15]. Three replicates of both focus groups were carried out in order to improve the richness of information that was collected. The inclusion criteria called for participants that were (1) aged above 21 and (2) not frequently consuming wholegrain foods. Individuals were excluded if they noted that they were wheat or cereal intolerant.

### 2.2. Focus Group Discussions

All participants completed two focus groups either side of a 14-day wholegrain product familiarization period to explore the attitudes of participants to these foods was assessed before and after this period in pre- and post-familiarization periods. As a number of subjects came from a degree programme where they studied nutrition, focus group participants were grouped according to degree background whereby two separate groups were conducted for participants “with nutrition knowledge” (i.e., studying nutrition-related subjects) and another focus group conducted for those without (studying other subjects). Each focus group discussion lasted approximately one hour. Focus group discussions were conducted in English and audio-recorded using a digital recorder (Olympus VN-702PC Digital Voice Recorder V405171SU000 (Tokyo, Japan) before being subsequently transcribed by a single researcher (JEN).

The structure of the initial (pre-familarisation) focus group included a discussion around barriers to whole grain consumption. They were also requested to share their attitudes towards 8 products which would be given to try during the familiarization period (within the focus group discussions and through Likert scale questionnaires—see below). The focus group also discussed exposure to and acceptability of wholegrain products that would be given to the participants in order to assess whether the 2-week familiarization period had changed their perceptions. Both focus groups included a section to evaluate the acceptability to the wholegrain products pre- and post-familiarization. Participants were required to indicate their attitudes to these products on the 4-point Likert scale, an ordinal scale of measurement. The response options were “never try”, “dislike”, “neutral” and “like”. The measurement of attitudes was effective to conclude the initial and post-attitudes using Likert scales [16].

The second focus group was a follow-up to discuss their experiences of products during the familiarization period. The impact of the whole grain familiarization period was measured by changes in liking of wholegrain products (also assessed via Likert scale questions).

### 2.3. Two-Week Wholegrain Product Familiarization Period

According to previous consumer studies, inferior taste had been the most salient barrier towards whole grain consumption [17] and product sampling has been shown to be effective in encouraging trying products for the first time [18]. Tasting actual products allows better discussion of an individual’s previous experience of the products [19].

During the familiarization period, a variety of wholegrain products were given to the participants to try. These products provided were brown rice, wholemeal pasta, wholemeal bread and oatmeal porridge, oatmeal cookies, granola bars, muesli and wheat biscuit-based ready-to-eat breakfast cereals. The respective wholegrain foods were chosen based on convenience, availability and variety. The selected items also contained a higher proportion of whole grains than the 51% that the US Food & Drug Administration have suggested for wholegrain foods [1]. The participants were provided with multiple servings of each product (at the end of the pre-familiarization focus group) as sold and asked to include these within their normal diet as they wished.

### 2.4. Qualitative Data Analysis

The number of respondent for each option in the questions was calculated with respect to the total number of respondents for the same question. All focus groups were transcribed into Word documents [20] and anonymised. These transcripts were then manually coded for common thematic elements whereby the data were analysed via constant comparison analysis given that there were several focus groups conducted within this study [21] by a single researcher (JEN). Similar themes were noted across all three groups.

### 2.5. Statistical Analysis

Mann-Whitney U tests were carried out to analyse whether whole grain acceptance was different pre- versus post-familiarization period using the Minitab software (version 17, Minitab Inc., Pennsylvania, PA, USA). Statistical significance was assumed if the *p*-value was less than 0.05.

## 3. Results

### 3.1. Perceived Barriers towards Whole Grain Consumption

**Figure 1:**
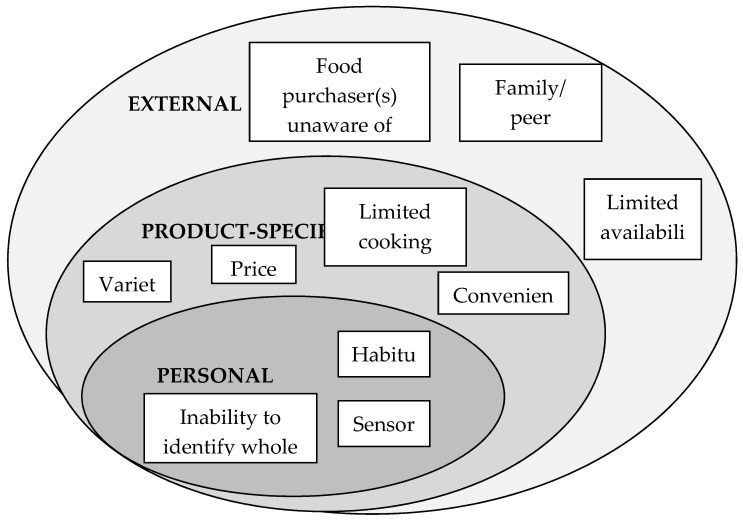
The barriers towards whole grain consumption in young Singaporean adults.

The focus group analysis resulted in the development of three key themes and putative explanations of barriers to whole grain consumption (see Figure 1). From focus group transcripts, “Sensory” and “Habitual” categories indicated to be the more frequent barriers with eight participants noting these among major barriers. Seven participants also noted “Family preference”, five noted “Availability” and two noted “Price” as potential barriers.

There was general agreement in all focus groups that eating habits developed from an early age would be a frequent barrier to future consumption. Individuals who are in their adulthood usually stick to their food consumption habits inculcated earlier in life. It would be difficult for them to change as they are not familiar to the taste and texture of the new food.

“*It is not a common practice for me to eat whole grains so I will eat whole grains only if there is (some present)…In a way, I can live or live without whole grains*.” (Participant #8 (female with nutrition knowledge), pre-familiarization focus group 2).

The percentage of males (27%, *n* = 3) who indicated “Sensory” categorised barriers as their prominent barrier was fairly similar to that of the females (26%, *n* = 5). A number of participants mentioned that although they are aware of health benefits associated with the product, they would not consider it during their subsequent purchase if the product is not to their personal liking.

“*I don’t like the wheaty taste of the whole meal products. Brown rice takes me a longer time to chew and I feel that it’s very important to enjoy food...This will result in wastage of money and I would not get the health benefits*.” (Participant #9 (male without nutrition knowledge), pre-familiarization focus group 3).

“*I just don‘t really like the grainy texture and floury taste of whole grains. I‘m the type of person who enjoys my food, so if I don’t like the taste of my food then I wouldn’t even bother purchasing it*.” Participant #5 (female with nutrition knowledge), pre-familiarization focus group 1).

The variety of products sold by manufacturing companies and restaurants might also have an influence on food choice. Participants suggested that they were more likely to select a range of products that were familiar to them.

“*There is limited availability of my favourite foods. For example: if they give me a whole grain alternative of my favourite cookie then I may consider trying out…if food companies continue producing foods that is not to my liking, I will definitely not buy them*!” (Participant #4 (female with nutrition knowledge), pre-familiarization focus group 1).

Price was mentioned in all the focus groups as a perceived barrier towards whole grain consumption. Participants consistently noted that wholegrain products generally cost more than refined products. Most participants could not relate why unprocessed products were actually more expensive.

“*If wholegrain products are more expensive than non-wholegrain products then I would rather eat non-wholegrain products. It would be very expensive to maintain the dietary habits of eating whole grain on a long-term basis.*” (Participant #10 (female with nutrition knowledge), pre-familiarization focus group 2).

The lack of cooking skills and experience of using wholegrain products were cited during discussion as potential barriers. Participants mentioned the issue of insufficient recipes and their lack of food preparation skills leading to frequent consumption of similar foods if whole grains were incorporated into the diet.

“*It is very likely that you consume the same dish over and over again if whole grains are consumed on a regular basis. Who would want to consume the same old boring dishes for every meal*?” (Participant #6 (male without nutrition knowledge), pre-familiarization focus group 3).

Preparation time was a recurrent barrier that was mentioned in the post-familiarization focus groups. Some wholegrain products that required long cooking time and extra preparation method, such as soaking of brown rice were considered to be more time-consuming or challenging in their preparation than non-wholegrain products.

“*Whole grains would require a longer time to prepare. For example: the texture of wholewheat pasta is slightly harder so it requires a longer cooking time.*” (Participant #6 (female with nutrition knowledge), pre- familiarization focus group 2.

However, more positive responses were focused around ready-to-eat wholegrain snacks. Participants mentioned that wholegrain snacks are items that are conveniently available them and could easily be consumed on-the-go.

“*Ready-to-eat wholegrain products don’t require much preparation. For example: the snack products (oatmeal cracker) and bread that you have actually showed at the start…we just need to buy the products and assemble.*” (Participant #6, (female with nutrition knowledge), pre-familiarization focus group 1).

All focus group participants lived with their family and their parents usually purchased food for the entire family. The food purchaser’s lack of ability to identify wholegrain products and lack of awareness of the potential health benefits were frequently cited, with parents/the food purchaser not being motivated to spend more of wholegrain products without being convinced of long-term health benefits.

“*I think that if we were to ask our parents about what is a whole grain, they would only be able to identify brown rice. They might not be able to relate cereals to whole grains. Besides that, they are not convinced that whole grains help to improve health, I had a hard time explaining to them and gave up after a while!*” (Participant #2 (female with nutrition knowledge), pre-familiarization focus group 1).

*“…our parents are the sole providers of the family and they are the ones who make the grocery purchasing. They made the food available for us, that‘s why it’s important for them to know the benefits in order for them to make the right decision*.” (Participant #8 (female with nutrition knowledge), pre-familiarization focus group 1).

Family preference for refined foods over wholegrain foods was a common barrier that was brought up during the discussion. It appeared that food preferences of every family member were taken into account especially for foods that would be cooked to share such as rice and pasta. The heads of the households tended to be the ones dictating their meals rather than the participants.

“*Most of the household groceries are purchased by my mum and she would cater to the majority preference. If I were to ask her to buy a wholegrain product, she would probably be against the idea...There is just a psychological barrier in them whereby they associate whole grains with the negative opinion on taste etc.*” (Participant #7 (female with nutrition knowledge), pre-familiarization focus group 2).

It was pointed out that peer preferences were problematic when eating out as a group. It was mentioned by one of the participants that it is quite challenging to convince peers to eat somewhere specifically based on wholegrain food availability.

“*If my friends or meal buddies chose to eat it in a place where they don‘t have whole grains…I wouldn’t get to eat whole grains. However, this is absolutely fine with me and I would go along with the majority as whole grain is not my No 1 choice of food!*” (Participant #10 (male without nutrition knowledge), pre-familiarization focus group 3).

### 3.2. Post-Familiarization Attitudes towards and Acceptance of Wholegrain Foods

All participants were able to try the oatmeal cookies, granola bars and wholewheat bread provided, while some participants did not try the other products provided (muesli *n* = 3, oatmeal porridge *n* = 8, wheat biscuit-based ready-to-eat breakfast cereal *n* = 8, whole wheat pasta *n* = 12 and brown rice *n* = 13). A Mann-Whitney U test was conducted to test for any significant difference in participants; acceptance of the products pre and post-familiarization, with possible Likert scale rankings of 1 = unwilling to try, 2 = dislike, 3 = neutral and 4 = like. If the generated median values were higher post-familiarization versus pre-familiarization, it would suggest an improvement in acceptance of these products.

Data in Table 1 highlight that there was a significant difference in the average acceptance scores of the wholegrain snack and staple products following the familiarization period (*p* < 0.05). A significant increase was seen in the median values of all wholegrain snacks (Oatmeal cookies, granola bar and muesli) from pre (1 in all cases) to post (4 in all cases, *p* < 0.01), although the increase for wheat biscuit breakfast cereal which has a lower increase in median scores from pre (1) to post (2, *p* = 0.0421). On the other hand, when comparing the individual staple products, only the wholemeal pasta acceptance significantly improved after the familiarization period (*p* < 0.05). Perceived acceptance of the wholemeal bread started off high and was did not change after the familiarization period. Within the focus groups after the familiarization period, most participants agreed that the taste and convenience of the wholegrain snacks was generally positive but texture was noted as an issue with the majority of wholegrain products. The idea that negative organoleptic qualities of some of the wholegrain items could be overcome by combining them with other foods was noted in relation as a means by which some participants were better able to incorporate the products into their diet.

“*I prepare the pasta with tomato-based (sauce) and I couldn’t tell any difference between the regular and the wholemeal pasta. It taste(s) awesome and I’m willing to opt for wholemeal pasta (in the future)!*” Participant #1 (male without nutrition knowledge), post-familiarization focus group 3.

“*The addition of raisin into the muesli made it acceptable with a tinge of sweet taste...I would definitely eat this in (the) future.*” Participant #2 (male without nutrition knowledge), post-familiarization focus group 3.

## 4. Discussion

### 4.1. Barriers to Wholegrain Food Consumption

The findings of this study largely confirm barriers to wholegrain food consumption noted in previous studies. However, due to the difference in cultural and geographical background of our participants versus those of previous research studies (mainly carried out in Europe and North America), this is stroking in itself. The present findings suggest that current dietary habit is a barrier to the majority of the participants and they may unconsciously stick to their previous eating patterns. Previous work on wholegrain food acceptance has also concluded that healthy or unhealthy eating habits from an early age might subsequently be translated to similar eating habits in adulthood [19]. The population of the current study represent a group of individuals who are transitioning to greater independence in many of the food choices outside of the family home [22]. This level of autonomy does not necessarily seem to be driving healthier food choices however and may be limited by lack of interest in adapting their previous dietary habit to incorporate more wholegrain foods [23].

These findings were not however in accordance with Kuznesof et al. (2012) [11] as habitual intake was not suggested to be a barrier within the older, UK-based sample of participants. Our study group was from a different cultural background and younger than the participants of Kuznesof et al., (2012). Another possible explanation for this difference could be the difference in the period over which participants got to try wholegrain foods and the approach taken. Within the current study, participants were requested to try and include the foods provided into their habitual diet but not to specific quantities, whereas participants within the study of Kuznesof et al., (2012) [11] had previously tried foods within a 16-week intervention where they were asked to consume target amounts (either 3 or 6 servings/day) of whole grain servings. Participants were required to consume a specific amount of servings in the over a longer period of time (16 weeks) for the whole grain interventions that preceded the evaluation of consumer perceptions in the Kuznesof et al. (2012) [11] study whereas the amount of required serving was not specified in this study and wholegrain products were only sampled over a 2-week period. The 16-week wholegrain food intervention (3 or more servings of whole grains to be consumed daily) previously noted resulted in a significant increase wholegrain food consumption in consumers with low habitual whole grain intake up to 1 year after the intervention had ceased [12]. However, within the current study, long-term changes to dietary habit were not assessed familiarization period. It seems plausible that an intervention with whole grains (or other food items not habitually and frequently consumed) for a longer time period would be likely to increase acceptability of such products. Future studies assessing the importance of time length of intervention and proportion of whole grains necessary to effect a long-term dietary change therefore seem pertinent.

Despite knowledge of potential benefits, consumers tend not to choose wholegrain foods due to their inferior taste, smell and appearance [24]. This is further affirmed by the findings from this study with around a quarter of participants listing sensory perception as a major barrier. Further from this, taste acceptability (or lack thereof) was frequently mentioned within focus groups. It must be noted that, although a number of qualitative and quantitative responses highlighted taste as a potential barrier, trialling the products tended to increase acceptability for most of the wholegrain foods provided. This suggests that direct familiarization with products (through including them in the diet for a short period of time) helps to overcome negative attitudes towards expected taste. The lower acceptability without prior familiarization may therefore be driven by the individual’s expectations of the product, possibly as a result of other cues.

The colour of foods could influence the desire to consume certain foods and visually appealing food would stimulate the appetite of an individual [25]. The “dull” brown colour of most whole grains was mentioned in this study as one of the reasons that may discourage individuals from consuming whole grains. These findings were consistent with previous qualitative research regarding the significance of sensory aspects in affecting food choices [26].

The results of this study suggest that there was a limited range of specific food products available to consumers in supermarkets, canteens, hawker centres and restaurants in Singapore. Previous US-based research has noted a similar lack of wholegrain food options in supermarkets [27]. Due to the frequency of out-of-home food consumption in Singapore, it therefore seems relevant to evaluate the ways in which a wider range of wholegrain products could be included into these outlets.

The higher perceived price of wholegrain foods was noted as being a limiting factor to out-of-home purchasing decisions. Most participants depend on their parents for a fixed weekly allowance. Current public health strategies within Singapore have tried to ensure that low-price vendors are subsided for offering healthier choices, including whole grain foods [28]. However, communication of this message (that wholegrain food options will not necessarily cost more) to members of the public appears to be key to ensuring improved adoption of dietary whole grains at a population level.

Food preparation and cooking skills tend to be lower among young adults [29]. Similarly, the participants in this study mentioned that they were not equipped with good culinary skills and may only cook on occasion. This may be especially challenging for cooking of wholegrain compared to non-wholegrain foods owing to the additional preparation method(s) or longer cooking times required [30]. The lack of proficiency in preparation of wholegrain foods was also suggested as a barrier to whole grain consumption in older adults who did not frequently cook in a similar previous study [11]. Our results and those of other groups therefore suggest that there may still be a need to develop individual‘s ability to prepare wholegrain foods in order to increase in-home consumption.

Most of the unmarried adults in Singapore continue to reside with their parents until they form their own family [31]. Participants noted that most of the parents/food preparers were not aware of the whole grain health benefits and suggested the need to educate the sole menu planner of the family. While education strategies aimed at children and young adults could be effective in altering their dietary intake in situations where they have purchasing decisions, it may not be ideal in altering dietary habit when at home and there appears to be a need to also develop interventions to benefit the delivery of healthier grain choices in the family home [32].

Eating meals as a group is an important cultural behaviour whereby social groups would have an impact on the selection of foods. Family/peer preference was nominated to be a major barrier compared to peer influences. The majority of Singaporeans appear to prefer refined grains to whole grains [7] and so this is likely to mean that choosing wholegrain food options as a group becomes less and less likely the larger the group size. This effect is also likely to impact on limiting wholegrain food intake within meals consumed as a family (either in-home or out-of-home). As eating as a group in Singapore and other parts of Asia frequently involves the practice of sharing large portions of communal foods [5], this may further exacerbate the tendency to choose refined grains.

Despite attempts to improve accessibility of whole grains in Singapore [28], limited availability was cited by many participants as a barrier to increased consumption and some voiced concerns that wholegrain alternatives were not widely available and they would not hesitate to consume whatever food that is readily available to them. This agreed with previous findings in a UK population [11] where participants often find it hard to comply with the recommended servings, especially when eating out-of-home, as refined grains were more accessible due to its high demand compared to whole grains.

The two-week product familiarization period significantly improved acceptability of most of the snack foods but only one of the staple food items. While snack items may be effective in improving intake of whole grains, they also tend to be higher in fat and sugar which may negate any individual- or population-level health benefits that increasing whole grain consumption could confer. Although wholemeal pasta and brown rice were amongst the least acceptable products, the general issue with not consuming these products was not mainly due to the inferior taste experience but family preferences and long preparation time which may have meant that the products were not sampled during the familiarization period. The grainy texture and off-putting colour of brown rice was denoted as inferior quality and would require a long and tedious process to convince the public of its acceptability [33]. The participants in the current study also noted that that grainy texture was a negative aspects which might offset product acceptability. Additionally, the feedback for the wheat biscuit breakfast cereal was poor with some participants totally put off by the product and would never want to purchase it again.

### 4.2. Strengths and Limitations of the Current Study

This study adopted a mixed qualitative and quantitative approach to evaluate in-depth information pertaining to the negative attitudes and behaviour towards whole grain consumption in young Singaporean adults. The selection criteria were targeted towards a group of individuals who were expected to be infrequent whole grain consumers. The focus groups were also relatively homogenous in their make-up, which it was hoped should improve frank and open discussion [20] and help engage all individuals in the discussion. As there are currently very limited data on the Asian consumer, focus groups are more appropriate to explore food beliefs and choices in much greater depth [21]. Due to the diverse perceptions of participants, the sample size does play a part in qualitative research. Repeated focus groups were carried out to minimise bias caused by a single observation, while the final total number of participants (*n* = 30) also allowed collection of a breadth of opinions. The study sample were not representative of the population of Singapore but represented a convenient group of individuals who were believed to have limited exposure to wholegrain foods despite high education levels.

Collection of detailed dietary data from participants (e.g. weighed food diaries or food frequency questionnaires) would have allowed evaluation of current whole grain consumption and other dietary habits within the participants and this could have been considered alongside quantitative and qualitative responses. Simple recipes of wholegrain foods which require preparation (brown rice, wholemeal pasta and oatmeal cereal) could have been provided to participants to increase the likelihood of trying the products during the familiarization period. Our research approach selected individuals who do not frequently consume whole grains. It would be particularly interesting to consider whether the attitudes of elective consumers of wholegrain foods are similar or divergent from non-consumers in future qualitative studies.

## 5. Conclusions

This study presented preliminary evidence that personal factors (habitual diet, sensory issues and inability to identify wholegrain products), product-specific factors (variety, price, lack of recipes and convenience) and external factors (parents unaware of health benefits, family or peer preferences and limited availability) are significant barriers to whole grain consumption. Amongst all the factors, sensory analysis and habitual dietary intake were concluded to be major barriers to increasing whole grain consumption. A short period of wholegrain product familiarization was not entirely successful in altering the taste preference of participants but it did manage to significantly improve the opinion of some wholegrain products and may be a means to encourage some individuals to incorporate them into their diets.

## Figures and Tables

**Table 1:** Acceptability of the wholegrain products provided pre and post-familiarization period.

Product	Caption	Median Score		
		Pre	Post	*p*-Value
Wholegrain Snacks	Oatmeal cookie	1	4	<0.001 *
	Granola bar	1	4	<0.001 *
	Muesli	1	4	<0.001 *
	WBBC	1	2	0.0421 *
Wholegrain staple	Oatmeal cereal	2	2	0.5997
	Brown rice	1	1	0.1087
	Wholemeal pasta	1	2	0.0046 *
	Wholemeal bread	4	4	0.4553

* *p*-value < 0.05 was considered to be statistically significant. WBBS = wheat biscuit breakfast cereal. 1 = unwilling to try, 2 = Dislike, 3 = Neutral, 4 = Like.
